# Effects of sesamol against acute kidney injury in cecal-ligation-and-puncture-treated rats

**DOI:** 10.1186/cc11757

**Published:** 2012-11-14

**Authors:** S Chien, Y Li, M Liu

**Affiliations:** 1 Tainan University of Technology, Tainan, Taiwan; 2 National Cheng Kung University, Tainan, Taiwan

## Background

Polymicrobial infection is associated with systemic inflammatory response, which is involved in the pathogenesis and development of acute kidney injury (AKI) [1,2]. In this study, we examined the effect of sesamol against AKI in cecal-ligation-and-puncture (CLP)-treated rats.

## Methods

Rats were given two subcutaneous doses of sesamol (10 mg/kg) 0 and 6 hours after CLP. Serum and kidney tissue were sampled 12 hours after CLP. Renal function and proinflammatory mediators, such as IL-1β, IL-6, and nitrite production were detected. Systemic oxidative stress was assessed by determining nitric oxide, superoxide anion, and xanthine oxidase activities. In addition, inducible nitric oxide synthase (iNOS) expression was also assessed in leukocytes from rats with AKI.

## Results

The levels of serum BUN, CRE, IL-1β, IL-6, nitrite, iNOS expression, superoxide anion, and xanthine oxidase activity were significantly higher in rats after CLP. Sesamol significantly inhibited all parameters in CLP-treated rats.

## Conclusion

Sesamol attenuated AKI by inhibiting neutrophil-initiated systemic inflammation and oxidative stress in CLP-treated rats.
